# Prostate Cancer: All Aspects

**DOI:** 10.1155/2013/265234

**Published:** 2013-10-01

**Authors:** Ahmet Tefekli, Murat Tunc, Volkan Tugcu, Tarık Esen

**Affiliations:** ^1^Bahcesehir School of Medicine, 34353 Istanbul, Turkey; ^2^Istanbul School of Medicine, Istanbul University, 34340 Istanbul, Turkey; ^3^Department of Urology, Bakırkoy Training and Research Hospital, 34360 Istanbul, Turkey; ^4^Koc University School of Medicine, 34365 Istanbul, Turkey

Prostate cancer is currently the most common cancer diagnosed in males living in developed nations and is becoming more commonly encountered in the rest of the world. This increase in the prevalence of prostate cancer is basically due to the advent of “Prostate Specific Antigen (PSA)” screening. However, an exciting debate and research are going around whether to screen or not, and more details are discussed in a review in this special issue. Diagnostic techniques have also evolved and have facilitated detection of majority of significant tumors. On the other hand, issues surrounding treatment are complex. Radical treatment options for prostate cancer are themselves not innocuous. However observing patients (active surveillance) carries its own risk of progression, especially because current methods of diagnosis do not distinguish aggressive from indolent tumors. Taking all these facts into consideration, it seems that much more effort will be spent on the research of prostate cancer in the near future. And therefore we decided to publish a special issue on prostate cancer, trying to cover all aspects of this important issue. Many papers especially from Asia and South America were submitted to this special issue and even only this demographic information is almost enough to underline the importance of prostate cancer as a growing health problem all over the world. And herein, outstanding findings of the published papers of this special issue are summarized.

In a timely interesting study from Austria by D. Junker et al., potentials and limitations of real-time elastography (RTE) for prostate cancer detection were assessed, considering the fact that cancers usually have a higher cell and vessel density than normal tissue and are therefore associated with a decreased elasticity. RTE findings were compared with whole-mount step section analysis of the prostate obtained from radical prostatectomy. RTE could detect 9.7% of cancer lesions with a maximum diameter of 0–5 mm, 27% of cancer lesions with a maximum diameter of 6–10 mm, and 70.6% of lesions with a maximum diameter of 11–20 mm while it could show 100% of lesions with a maximum diameter of >20 mm. In addition, there was a significant higher rate of cancer detection rate in those with predominant Gleason pattern 4 or 5 regarding cancer lesions with a volume ≥0.2 cm^3^. And the authors further suggest that adding information about contrast media dynamics in a multiparametric way might decrease the number of false negative cases in the ultrasonic evaluation of prostate cancer.

Several preoperative and postoperative nomograms, most of them originating from western countries, are being used in order to predict the outcome of prostate cancer, and many validations studies have been published in the literature. In an interesting study by V. H. W. Yeung et al. from Hong Kong, predictive accuracy of Kattan and Stephenson nomograms in the Chinese population was investigated for the first time in the literature. Despite a limited number of patients, the authors could observe that the 5-year and 10-year biochemical free survival rates in Chinese patients were similar to the predicted values by the Kattan and Stephenson nomograms.

Almost 90% of prostate cancer cases diagnosed in the era of PSA are believed to be “organ confined,” and “radical prostatectomy (open or laparoscopic/robot assisted)” is conserved to be the treatment of choice in the majority of cases. There have been a tremendous number of publications in the last decade about laparoscopic radical prostatectomy, investigating the outcome, learning curve, and almost all other aspects. But probably nobody has ever dared to analyze or publish the learning curve of a low volume surgeon. A. I. Mitre et al. from Brazil investigated this common but “never asked” question. The authors conducted a prospective study on 165 patients operated over an 8-year period by a single surgeon with previous laparoscopic experience. Sequential analyses were performed and, in order to define the learning curve, patients were divided into 3 groups of 55 patients arranged in chronological order. The results showed that intraoperative complications and conversions to open surgery were significantly less after the first 51 cases. All other parameters (blood loss, operative time, and positive surgical margins) significantly decreased and stabilized after 110 cases. Although there are several limitations in the study, the authors have to be congratulated for their efforts to collect and report their results.

Radical prostatectomy is nowadays the treatment of choice in the management of organ confined prostate cancer, and biochemical failure, reported to develop 20–30%, is becoming more commonly encountered. N. P. Murray et al. from Chile examined the presence of circulating prostate cells in blood after radical prostatectomy, using standard immunocytochemistry with anti-PSA monoclonal antibodies. They reported that circulating prostate cancer cells in blood were detected more frequently in patients with positive margins, capsular invasion, and vascular and lymphatic infiltration. They also concluded that presence of circulating prostate cancer cells was an independent risk factor associated with biochemical recurrence.

There are also several research studies published in this special issue. In a very interesting cell culture study submitted from Baltimore, USA, by A. Gupta et al., researchers examined the biological consequences of matrix metalloproteinase 9 (MMP9) knockdown in the invasion of prostate cancer (PC3) cells. It has been previously shown that MMP9 localized in invadopodia facilitates extracellular matrix degradation and invasion in PC3 human prostate carcinoma cells, by switching CD44 isoform expression from CD44 standard to CD44v6, which may be essential for the protection of noninvasive cellular phenotype. Although there are conflicting results regarding expression of CD44 and tumor characteristics, the researchers were the first to show that MMP9 knockdown increased CD44v6 expression and suggest that interaction between CD44 and MMP9 is a potential mechanism of invadopodia formation in PC3 cells. They also postulate that CD44v6 may be a potential marker for prognosis.

In another cell line study (LNCaP cell line) by S. S. Kim et al. from Korea, factors related to the development of androgen independent prostate cancer were investigated. High passage subcultured LNCaP cells acquired androgen independent property and the silencing of androgen receptor (AR) with small interfering RNA (siRNA) transfection resulted in the reversion of proteomic profile to level of fresh cell line. Furthermore, the expressions of five cancer related proteins (AR, heat-shock protein 27, clusterin, glucose-related protein 78, and cellular FLICE-like inhibitory protein) were increased in late stage (over 81 times subcultured LNCaP cell line) LNCaP. However these cancer related protein expressions were reversed with small interfering RNA (siRNA) transfection. These findings support that therapeutic approaches targeting AR can enhance the efficacy of anticancer treatment in the patients with castration resistant prostate cancer.

In another research study using PC3 cell line, S. S. Kim et al. from Korea investigated the change of doxazosin induced apoptosis after dual gene silencing of heat-shock protein 27 and cellular FLICE-like inhibitory protein (c-FLIP) in PC-3 cancer cells. They elegantly showed that dual silencing using siRNAsis technically feasible and knock out of c-FLIP and Hsp27 gene together enhances apoptosis with doxazosin in PC-3 cells. This finding suggests a new strategy of multiple knockout of antiapoptotic and survival factors in the treatment of late stage prostate cancer refractory to conventional therapies.

And finally, the guest editors of this special issue summarized the future prospects in the management of localized prostate cancer. As a conclusion, prostate cancer is a wide fertile area for both basic and clinical research and revolutionary changes in the diagnosis and management of prostate cancer are awaited in the near future.

*Ahmet Tefekli* *Ahmet Tefekli* *Murat Tunc* *Murat Tunc* *Volkan Tugcu* *Volkan Tugcu* *Tarık Esen* *Tarık Esen*

